# Factors Associated to Endemic Dental Fluorosis in Brazilian Rural Communities

**DOI:** 10.3390/ijerph7083115

**Published:** 2010-08-06

**Authors:** Efigênia F. Ferreira, Andréa Maria D. Vargas, Lia S. Castilho, Leila Nunes M. Velásquez, Lucia M. Fantinel, Mauro Henrique N. G. Abreu

**Affiliations:** 1 Department of Community and Preventive Dentistry, Universidade Federal de Minas Gerais, Avenida Antônio Carlos, 6627–31270.901 sala 3304, Belo Horizonte, MG, Brazil; E-Mails: efigeniaf@gmail.com (E.F.F.); vargasnt@task.com.br (A.M.D.V.); 2 Department of Operative Dentistry, Universidade Federal de Minas Gerais, Avenida Antônio Carlos, 6627–31270.901, Belo Horizonte, MG, Brazil; E-Mail: liacastilho@ig.com.br; 3 Department of Geology, Universidade Federal de Minas Gerais, Avenida Antônio Carlos, 6627–31270.901, Belo Horizonte, MG, Brazil; E-Mails: menegase@yahoo.com.br (L.N.M.V.); luciafantinel@gmail.com (L.M.F.)

**Keywords:** dental fluorosis, epidemiology, geology, multilevel analysis

## Abstract

The present paper examines the relationship between hydrochemical characteristics and endemic dental fluorosis, controlling for variables with information on an individual level. An epidemiological survey was carried out in seven rural communities in two municipalities in the state of Minas Gerais, Brazil. The Thystrup & Fejerskov index was employed by a single examiner for the diagnosis of dental fluorosis. A sampling campaign of deep groundwater in the rural communities of interest was carried out concomitantly to the epidemiological survey for the determination of physiochemical parameters. Multilevel modeling of 276 individuals from seven rural communities was achieved using the non-linear logit link function. Parameters were estimated using the restricted maximum likelihood method. Analysis was carried out considering two response variables: presence (TF 1 to 9) or absence (TF = 0) of any degree of dental fluorosis; and presence (TF ≥ 5—with loss of enamel structure) or absence of severe dental fluorosis (TF ≤ 4—with no loss of enamel structure). Hydrogeological analyses revealed that dental fluorosis is influenced by the concentration of fluoride (OR = 2.59 CI95% 1.07–6.27; p = 0.073) and bicarbonate (OR = 1.02 CI95% 1.01–1.03; p = 0.060) in the water of deep wells. No other variable was associated with this prevalence (p > 0.05). More severe dental fluorosis (TF ≥ 5) was only associated with age group (p < 0.05). No other variable was associated to the severe dental fluorosis (p > 0.05). Dental fluorosis was found to be highly prevalent and severe. A chemical element besides fluoride was found to be associated (p > 0.05) to the prevalence of dental fluorosis, although this last finding should be interpreted with caution due to its *p* value.

## 1. Introduction

When consumed in small doses, fluoride continually contributes toward the reduction in the prevalence and progression of dental caries. However, ingestion may cause changes in the tooth enamel, namely, dental fluorosis [1]. Individuals who permanently live in areas with water sources that have high concentrations of this element may ingest it in excess, the result being the emergence of what is commonly called endemic dental fluorosis [1,2], as reported for different parts of the world [3]. The prevalence and severity of dental fluorosis depend, among other factors, on the time of exposure and level of fluoride ingested [4].

Fluoride is widely distributed in combination with various minerals that occur naturally in the Earth’s crust, especially fluorite. Under normal circumstances, surface waters contain fluoride concentrations below 1.0 mg/L. However, depending on the geological conditions, groundwater has a greater likelihood of having contact with large amounts of fluorite. Fluoride levels tend to be higher in alkaline waters and geothermal areas. Calcium ions occur in excess in a large number of aquifers and, under such conditions, the concentration of fluoride is controlled by CaF_2_ [5]. Fluoride concentration has also demonstrated correlations with pH and concentrations of bicarbonates, calcium, magnesium and sodium [6,7].

Due to the scarcity of surface water in the rural regions of the municipalities of São Francisco and Verdelândia in Brazil, there was a need to sink deep tubular wells at the end of the 1970s to ensure the public water supply. Since the 1990s endemic dental fluorosis has been diagnosed in a number of rural communities in these municipalities [8,9].

Despite the identified correlations between fluoride and the hydrochemical characteristics of public drinking water, there is a gap in the scientific literature regarding the impact of these correlations on the human organism, especially dental fluorosis. While the association between fluoride concentration in the public water supply and dental endemic fluorosis is well established [4,10,11], it is necessary to determine whether there is an impact from other hydrochemical characteristics in the water supply on dental fluorosis in endemic areas.

The present paper examines the relationship between hydrochemical characteristics and dental fluorosis, controlling for individual demographic variables and access to dental treatment and employing multilevel analysis techniques. The article assesses the magnitude of the variation in dental fluorosis that can be explained by individual and hydrochemical characteristics.

## 2. Experimental Section

### 2.1. Study Site

**Figure 1.**
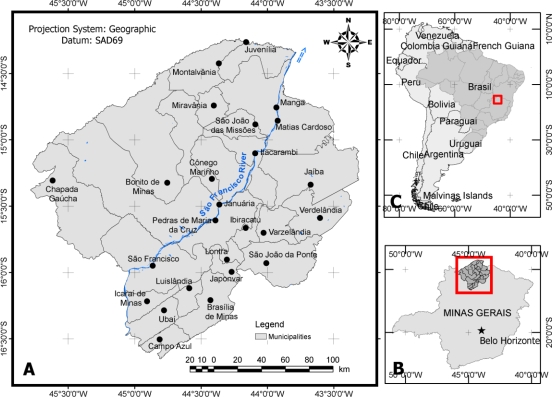
**(a)** Map showing the study area (São Francisco and Verdelândia). **(b)** Map showing the northern portion of Minas Gerais. **(c)** Location of Minas Gerais in Brazil.

The municipalities of São Francisco and Verdelândia are located in the northern portion of the state of Minas Gerais, Brazil, in the mid hydrographic basin of the São Francisco River (Figure 1). The cities pertain to one of the poorest regions in the state, the socioeconomic context of which is aggravated by the limited availability of surface water in rural areas, as well as a lack of the integrated planning and management of water resources.

The area has a semi-humid, warm, tropical climate [12]. The mean annual temperature is 24 °C in São Francisco and 25 °C in Verdelândia, with mean maximal temperatures of 32.3 °C and 30.8 °C, respectively. Mean rainfall is 1,132.9 mm/year in São Francisco and 789.8 mm/year in Verdelândia. Rains are distributed among the four months of summer, followed by a long dry period that further aggravates the problem of water availability [13]. In rural communities, the water supply is mainly ensured through groundwater, the access to which is attained through tubular wells.

The geological framework is represented (from the base to the top) by horizontal and sub-horizontal layers of calcareous and pelitic rocks (Bambui group), arenite (Urucuia Group) and sediment. These pockets of rock and sediment define two water-connected hydrogeological systems: the karstic-fissure system of the base and the overlaid granular system.

### 2.2. Epidemiological Survey

This study received approval from the Human Research Ethics Committee of the Federal University of Minas Gerais (Brazil). An explanation was given to parents/guardians regarding the objectives and methods of the study. Anonymity was ensured for the subjects. All doubts were clarified before initiating the study and parents/guardians of the children examined signed informed consent forms.

In the municipality of São Francisco, epidemiological oral health surveys were carried out in the rural communities of Mocambo, Vaqueta and Novo Horizonte in 2002 and in the communities of Barreiro dos Anjicos, Brejo dos Anjicos and Furado Grande in 2007. The district of Amargoso, located in the municipality of Verdelândia, participated in a study in 2005.

The population selected for the present study was made up of lifelong residents between six and 22 years of age. The age of six years was chosen due to the beginning of the change in dentition. The upper limit age of 22 years was chosen because individuals above this age may not have been exposed to the excessive ingestion of fluoride from well water during the formation of their dental enamel. There is little information on the target population—children living in one of seven rural communities in the two municipalities. The overall 6-to-22-year-old population was estimated at 22,175 in the whole of São Francisco and 3,077 in the whole of Verdelândia. However, this information is less useful, as the number of people that lived in each rural community was unknown. Considering this limitation, the present convenience sample was selected with the help of representatives of the respective city halls and community leaders. The latter discussed and explained the objectives of the study in each rural community and scheduled visits for data collection. Most of the subjects were residents in the countryside and lived far from each other. On the previously scheduled day, fireworks were used to call the population for the exams. All individuals who appeared at the predetermined locations (schools, healthcare units) and gave informed consent were examined.

The clinical examinations were carried out by a single, previously trained examiner with excellent intra-examiner agreement (kappa = 0.95) under natural light, with prior plaque removal using a toothbrush. Intra-examiner reproducibility was calculated on a tooth-by-tooth basis. All teeth were dried with gauze for the exam. No dental instruments (mirror, explorer) were used. The TF index was used for the diagnosis of dental fluorosis, examining all surfaces of all teeth, with each tooth scored based on the most affected surface. Scores ranged from 0 (absence of dental fluorosis) to 9 (severe dental fluorosis). A score of zero denotes enamel with normal translucency. Increasing values on the ordinal scale of the index denote an increase in the severity of dental fluorosis. Scores 1 to 4 denote increasing degrees of opacity with no loss of the outermost enamel. Scores of 5 or more denote increasing degrees of loss of the outermost enamel. This index is capable of detecting different aspects of the severity of dental fluorosis in areas that have a public water supply with high concentrations of fluoride [4,14]. No individuals wore orthodontic appliances. Teeth with dental restorations (n = 2) were not examined. The diagnosis of dental fluorosis was based on a pattern of white opacities, with a diffuse distribution over the surface of varying intensity and other characteristics. Opacities may vary from minor white striations and lines to small or extensive areas of opaque enamel. Loss of the outermost enamel (pits) and staining of the enamel were also considered. Homologous teeth are affected, but not all homologous teeth are affected identically [4,15].

### 2.3. Geological/Hydrogeological and Hydrochemical Studies

Stratigraphic units were mapped and mineralogical identifications were carried out using electron microscopy and x-ray diffraction. Hydrogeological studies began with the analysis of records on deep wells. A sampling campaign of deep groundwater in the rural communities of interest was carried out concomitantly to the epidemiological survey, measuring the following physical parameters *in situ*: pH, electrical conductivity and total dissolved solids. The following were determined in the laboratory: F^−^, Ca^2+^, Na^+^, K^+^, Mg^2+^, Cl^−^ and HCO_3_^−^. Analytical, water collection and water preservation procedures were carried out in compliance with the Standard Methods for the Examination of Water and Wastewater, 20th edition [16]. These hydrochemical characteristics were the ecological variables of the study.

### 2.4. Statistical Analysis

**Figure 2.**
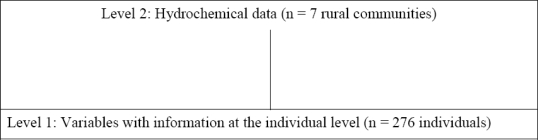
Hierarchical structure of hydrochemical data and variables with information at the individual level.

The nature of the data, which involved variables with information on the individual and ecological level, and the hypotheses involved suggest the need for multilevel analysis. Two databanks—one for variables with information on the individual level and another for hydrochemical variables—were constructed using the Statistical Package for Social Sciences (SPSS for Windows version 17.0). The multilevel modeling of the 276 individuals in seven rural communities was achieved through the non-linear logit link function. Parameters were estimated by the restricted maximum likelihood method using the HLM 6.06 statistical package. Analysis was carried out considering two response variables: presence (TF 1 to 9) or absence (TF = 0) of any degree of dental fluorosis; and presence (TF ≥ 5—with loss of enamel structure) or absence of severe dental fluorosis (TF ≤ 4—with no loss of enamel structure). The worst tooth score was used for measuring TF of each individual. Both response variables were binary categorical variables. Dummy variables were established for the age groups. The study had two major objectives. The first was to examine the extent to which hydrochemical characteristics added an independent contribution to the prediction of dental fluorosis when controlling for individual characteristics. For such, the modeling strategy was geared toward testing whether hydrochemical characteristics offered a significant improvement in predicting dental fluorosis over and above the prediction achieved by individual demographic characteristics and access to dental treatment. The rationale for adopting this strategy was to establish that the effects of hydrochemical characteristics on dental fluorosis were unique and not merely a reflection of some shared variance between this measure and other individual characteristics. Only then is the inclusion of hydrochemical characteristics in the model warranted and the use of multilevel modeling required. The second objective was to assess the role of individual demographic characteristics and access to dental treatment in explaining the observed variation in dental fluorosis rates within and between rural communities. To achieve the study goals, a multilevel model (Figure 2) was constructed. The baseline model did not include any covariates. This intercept-only model was the so-called “null model”.

Differences in dental fluorosis here depend only on the mean difference for all individuals in all groups as well as a difference for each group and each individual. The Level 1 variables were incorporated into the model one by one, with the calculation of p-values (Student’s t-test). In cases of an association between a Level 1 variable and the response variable, it was determined whether there were differences in this influence for each of the communities. After the selection of the Level 1 variables, the hydrochemical variables (Level 2) were incorporated one by one. Considering the plausibility of an association between fluoride and dental fluorosis, each model was constructed maintaining the Level 2 variable *fluoride*. Odds ratios (OR) and 95% confidence intervals (95%CI) were calculated for each Level 1 and Level 2 co-variable. The reliability estimate was used to determine the adequacy of the final multilevel model [17].

## 3. Results and Discussion

### 3.1. Results

The majority of individuals surveyed were male (51.1%) and had no access to dental care (66.7%). Ninety-nine children (35.9%) were 6 to 9 years of age; 92 (33.3%) were between 10 and 12 years; 64 (23.2%) were adolescents between 13 and 15 years; and 21 (7.6%) were between 16 and 22 years of age. Dental fluorosis was found in 222 individuals (80.4%) and 135 individuals (48.9%) exhibited severe dental fluorosis (TF ≥ 5; Table 1).

Table 2 displays the hydrochemical data of the different communities. The high degree of electrical conductivity indicates relatively salinized waters, with total dissolved solids (TDS) ranging from 273 to 687 mg/L. With the exception of fluoride, the ions listed in the table are responsible for this salinization. The pH values denote high alkalinity of the waters.

The analysis of the final estimation of variance components of the null model revealed that the prevalence of dental fluorosis was different between the different rural districts of São Francisco and Verdelândia (p < 0.001), thereby corroborating the need for the use of a multilevel model for the analysis of the variables.

Analyzing the factors associated with dental fluorosis, the inclusion of any Level 1 variable (age group, gender and access to dental care) led to no significant improvement in the fit of the model (p > 0.05). Among the hydrochemical characteristics, an increase of one unit in the concentration of fluoride led to a 2.59-fold increase in the chance of an individual having any degree of dental fluorosis (95%CI: 1.07–6.27; p = 0.073), whereas an increase of one unit of bicarbonate led to a 1.02-fold increase in the chance of an individual having any degree of dental fluorosis (95%CI: 1.01–1.03; p = 0.060) (Table 3). Although CI95% did not included the unit, these results should be analyzed with caution considering that *p* value is higher than 0.05. The reliability estimate for this model was 0.716.

The analysis of the final estimation of variance components of the null model revealed that the prevalence of TF ≥ 5 dental fluorosis was different between the different rural communities of São Francisco and Verdelândia (p < 0.001), once again corroborating the need for the use of a multilevel model for the analysis of the variables.

An analysis of the associated factors revealed that age alone was associated to the prevalence of severe dental fluorosis. In the final model, in relation to children six to nine years of age, those between 10 and 12 had a 1.95-fold greater chance of having severe dental fluorosis (95% CI: 1.03–3.69; p = 0.040) and those between 13 and 15 years had a 3.10-fold greater chance of having severe dental fluorosis (95% CI: 1.41–6.85; p = 0.006). In the comparison between the youngest children and individuals between 16 and 22 years of age, both the 95%CI (0.93–8.18) and the level of statistical significance (p = 0.067) indicate an absence of an association (Table 4). Based on the results of the final estimation of variance components, the influence of age on the prevalence of severe dental fluorosis did not vary between communities (p > 0.05). The reliability estimate for this model was 0.888.

### 3.2. Discussion

The context of the present study involves rural communities with a history of water scarcity and policies regarding access to water resources that, until very recently, were inadequate. Regarding the natural occurrence of fluoride in the water supply, only Furado Grande had a concentration below the limit proposed by the World Health Organization of 1.5 mg/L [3]. However, based on the climatic characteristics of the rural communities investigated, the recommended safe range would be from 0.7 to 0.8 mg/L [18]. Thus, both the prevalence and severity of dental fluorosis in these communities are high. In Estonia, the prevalence of dental fluorosis in areas with concentrations below 1.0 mg/L was found to be 6.7%, whereas twice this value was found in areas in which the concentration reached up to 1.5 mg/L and the risk of dental fluorosis was found to be 4.4-fold greater in areas in which the concentration ranged from 1.5 mg/L to 2.0 mg/L [1].

The prevalence of dental fluorosis was associated to the concentration of fluoride in the water supply. Geological studies identified an important origin of fluoride in the groundwater of karstic-fissure aquifers that stems from the dissolution of fluorite found in calcareous rock. Over time, the contact between water and rock (partially a result of the low local precipitation) is believed to be responsible for the increase in the concentrations observed at different sites in the area, especially in locations of aquifer discharge [13].

Besides the concentration of fluoride, the present study identified another hydrochemical characteristic that increased the prevalence of dental fluorosis. Although not reported in the literature consulted, the relation between dental fluorosis and bicarbonate is plausible, as the availability of fluoride is increased by the bicarbonate content in the water supply [6,19]. Rao [20] pointed out a possible dissolution of fluorite with a simultaneous precipitation of CaCO_3_, which would explain the negative relation between fluoride and calcium and the positive relation with sodium. Sodium bicarbonated waters are effective in making F^−^ available from fluorite. The same author illustrates the process by means of the following reactions:

(a) $${\text{CaF}}_{2}\;+\;{\text{Na}}_{2}{\text{CO}}_{3}\;\Leftrightarrow {\text{CaCO}}_{3}\;+\;2\text{Na}\;+\;2\text{F}$$

(b) $${\text{CaF}}_{2}\;+\;{2\text{NaHCO}}_{3}\;\Leftrightarrow \;{\text{CaCO}}_{3}\;+\;2\text{Na}\;+\;2\text{F}\;+\;{\text{H}}_{2}\text{O}\;+{\text{CO}}_{2}$$

The origin of carbonates and sodium bicarbonates is the alteration in the clay and micaceous minerals of pelites, which, according to Rao [20], cause reactions “a” and/or “b”, thereby making sodium and fluoride available. As both are very soluble ions, their concentration is largely favored. Sodium bicarbonated waters, which are a significant characteristic of the rural communities surveyed, are effective at making F^−^ available from fluorite [20].

The involvement of F^−^ with HCO_3_^−^ is nearly linear. The high content of bicarbonate in the water gives it an alkaline nature, which favors the stability and mobility of F^−^ ions in groundwater. The results of the regression analysis on the dependence of F^−^ and HCO_3_^−^ (total alkalinity) indicate a strong, positive correlation, with the simultaneous release of hydroxyl and bicarbonate ions during processes of lixivium and dissolution of the fluoride content in groundwater [21].

The other hydrochemical characteristics in the deep wells do not appear to be associated to the increase in the prevalence of dental fluorosis. From the literature consulted, this concentration of fluoride is also affected by pH [5–7,19]. In the present study, however, no such association was found. Despite the expected correlation between fluoride concentration in the water supply and calcium [7], sodium [21] and other cations [22,23], there was no impact from these elements on dental fluorosis in the present study. It should also be stated that the present study measured dental fluorosis and exposure at the same time.

Due to the impossibility of using a random sample, a convenience sample was used. The number of examined subjects and the age distribution were different in each rural community. Despite the efforts on covering the target population, our sampling methodology may have influenced our results. The cross-sectional study design has also limitations regarding explanations about the causality of any disease. The ecological fallacy cannot be disregarded in the findings of this study. Although the options were residual, some individuals may have also had access to uncontaminated water during the period of tooth formation. Moreover, the concentration of fluoride and other ions in the water supply is highly variable in this region. Finally, the existence of correlations between hydrochemical characteristics does not necessarily ensure the impact of these characteristics on dental fluorosis.

In the present study, the concentration of fluoride was not associated to the severity of dental fluorosis (TF ≥ 5). Recent studies have identified a greater severity of dental fluorosis in areas with high fluoride content when compared to areas with lower concentrations [10,11]. As the present study compared communities that used water with a fluoride concentration above the recommended levels at some time in recent history, it would be difficult to find an association when exposure to the water is homogeneously inadequate.

According to the literature consulted, boys are more often affected by severe dental fluorosis than girls [10,24]. However, no statistical differences between genders were found in the present study or in a study carried out by Saravanan *et al*. [25]. Likewise, access to dental care could have exposed individuals to the professional use of fluoride. Moreover, access to a dentist could have protected individuals through early diagnosis. In this case, dentists may have given information on the consumption of water without high levels of fluoride. However, this variable had no influence over the condition investigated. Access to dental care among a minority of the individuals surveyed was not regular, as public services in rural areas were residual and unorganized in the years surveyed.

We did not evaluate other additional sources of fluoride during infancy, as toothpaste or industrialized foods. Despite considering that these factors are important in etiology of dental fluorosis [26,27], the consumption of these foods is, probably smaller in São Francisco and Verdelândia than in urban regions. Moreover, the cities pertain to one of the poorest regions in the state and, the consumption of toothpaste in childhood may be not relevant for endemic dental fluorosis [28].

The prevalence of more severe dental fluorosis (TF ≥ 5) was similarly influenced by age in the different communities surveyed. As demonstrated in a previous study [29], the prevalence and severity of dental fluorosis is directly related to the amount of fluoride ingested, age at the time of exposure and duration of exposure. The highest prevalence of severe dental fluorosis was observed among individuals between 13 and 22 years of age. The fewer fully erupted teeth among younger children may explain this finding. Moreover, two rural communities in the municipality of São Francisco stopped consuming water with high fluoride levels in 1995 and 1996. Thus, children who were 6 to 9 years old (n = 36) in 2002 (year of the epidemiological survey) in these two communities had had fewer years of exposure to fluoride. Besides, TF index gets worse as the more posterior teeth erupt and become subjected to occlusal forces which lead to surface breakdown of the fluorotic enamel.

## 4. Conclusions

Dental fluorosis in the regions studied is highly prevalent and severe. A chemical element besides fluoride was found to be associated (p > 0.05) to the prevalence of dental fluorosis. This last finding should be interpreted with caution, however, due its *p* value. Age was the only factor associated (p < 0.05) to the severity of dental fluorosis. Further studies on the impact of the hydrochemical characteristics of drinking water on human health should be carried out in locations with distinct climatic, geological and geographical characteristics.

## Figures and Tables

**Table 1.** Individual demographic characteristics, access to dental treatment and dental fluorosis in different rural communities, São Francisco and Verdelândia, Brazil.

**District**	**Access to dental care**	**Proportion of females**	**Age groups (years)**				**TF = 0**	**TF 1 to 4**	**TF ≥ 5**
			**6 to 9**	**10 to 12**	**13 to 15**	**16 to 22**			
Mocambo (n = 65)	43.1%	44.6%	30.8%	33.8%	21.5%	13.8%	20.0%	27.7%	52.3%
Vaqueta (n = 47)	14.9%	42.6%	46.8%	34.0%	8.5%	10.6%	6.4%	27.6%	66.0%
Novo Horizonte (n = 46)	32.6%	52.2%	34.8%	28.3%	23.9%	13.0%	2.2%	19.5%	78.3%
Amargoso (n = 57)	40.4%	54.4%	29.8%	43.9%	24.6%	1.8%	8.8%	45.6%	45.6%
Barreiro Anjicos (n = 33)	45.4%	48.5%	15.2%	21.2%	63.6%	0.0%	81.8%	9.1%	9.1%
Brejo do Anjicos (n = 13)	15.4%	53.8%	84.6%	15.4%	0.0%	0.0%	23.1%	53.8%	23.1%
Furado Grande (n = 15)	13.3%	53.3%	53.3%	40.0%	6.7%	0.0%	13.3%	73.4%	13.3%
Total (n = 276)	33.3%	51.1%	35.9%	33.3%	23.2%	7.6%	19.6%	31.5%	48.9%

**Table 2.** Hydrochemical characteristics in different rural districts, São Francisco and Verdelândia, Brazil.

**Communities**	**Fluoride (mg/L)**	**Calcium (mg/L)**	**Bicarbonate (mg/L)**	**Sodium (mg/L)**	**Chloride (mg/L)**	**Magnesium (mg/L)**	**pH**	**Conductivity**
Mocambo (n = 65)	3.2	80.9	375.8	95.0	20.9	9.7	7.60	961.8
Vaqueta (n = 47)	3.0	176.7	375.5	185.0	381.0	5.6	7.80	1,195.2
Novo Horizonte (n = 46)	3.9	23.1	363.0	170.0	121.5	6.1	8.50	1,202.9
Amargoso (n = 57)	4.8	170.4	351.4	28.7	169.0	14.7	7.79	1,084.0
Barreiro Anjicos (n = 33)	2.2	28.5	243.4	235.0	283.0	4.7	8.50	1,621.3
Brejo do Anjicos (n = 13)	2.6	23.9	452.5	195.0	98.0	10.8	8.00	942.1
Furado Grande (n = 15)	1.4	22.9	473.0	62.7	4.4	53.3	8.00	503.9

**Table 3.** Multilevel models for variables associated to the presence or absence of dental fluorosis in rural communities of São Francisco and Verdelândia, Brazil.

**Models with each Level 1 variable**		OR (95%CI)	Standard Error	p-value

Age (years)	6 to 9	1.00	0.6148	
	10 to 12	1.72 (0.68–4.40)	0.4764	0.255
	13 to 15	0.92 (0.34–2.47)	0.5042	0.864
	16 or more	1.55 (0.30–8.05)	0.8367	0.599

Gender	Female	1.00	0.6254	
	Male	1.13 (0.55–2.34)	0.3694	0.742

Access to dental care	No	1.00	0.6202	
	Yes	0.65 (0.30–1.40)	0.3882	0.269

**Models with Level 2 variables**				

Fluorine		2.07 (0.58–7.40)	0.5744	0.262

Fluorine		1.91 (0.34–10.84)	0.7862	0.457
Calcium		1.00 (0.98–1.03)	0.0124	0.875

Fluorine		2.59 (1.07–6.27)	0.4002	0.073
Bicarbonate		1.02 (1.01–1.03)	0.0059	0.060

Fluorine		1.79 (0.74–4.35)	0.6478	0.221
Sodium		0.99 (0.98–1.01)	0.0093	0.443

Fluorine		2.15 (0.53–8.67)	0.6309	0.292
Chlorine		1.00 (0.99–1.01)	0.0050	0.634

Fluorine		2.98 (0.71–12.48)	0.6491	0.167
Magnesium		1.05 (0.95–1.16)	0.0433	0.330

Fluorine		1.95 (0.48–7.97)	0.6372	0.354
pH		0.43 (0.01–36.84)	2.0171	0.695

Fluorine		2.54 (0.88–7.37)	0.4822	0.123
Conductivity		1.00 (0.99–1.00)	0.0015	0.135

**Table 4.** Multilevel models for variables associated to the presence or absence of TF ≥ 5 dental fluorosis in rural communities of São Francisco and Verdelândia, Brazil.

**Models with each Level 1 variable**		OR (95%CI)	Standard Error	p-value

Age (years)	6 to 9	1.00	0.5118	
	10 to 12	1.95 (1.03–3.69)	0.3242	0.040
	13 to 15	3.10 (1.41–6.85)	0.4027	0.006
	16 or more	2.76 (0.93–8.18)	0.5525	0.067

Gender	Female	1.00	0.4870	
	Male	0.90 (0.54–1.51)	0.2644	0.689

Access to dental care	No	1.00	0.4866	
	Yes	0.88 (0.50–1.54)	0.2868	0.650

**Models with Level 2 variables**				
Age (years)	6 to 9	1.00	0.4549	
	10 to 12	1.93 (1.02–3.66)	0.3251	0.044
	13 to 15	3.02 (1.37–6.64)	0.4012	0.007
	16 or more	2.72 (0.92–8.07)	0.5522	0.070
Fluorine		2.14 (0.80–5.69)	0.4437	0.147

Age (years)	6 to 9	1.00	0.5238	
	10 to 12	1.94 (1.03–3.68)	0.3252	0.042
	13 to 15	3.13 (1.41–6.93)	0.4042	0.006
	16 or more	2.74 (0.92–8.12)	0.5527	0.069
Calcium		1.01 (0.99–1.03)	0.0079	0.378

Age (years)	6 to 9	1.00	0.5446	
	10 to 12	1.97 (1.04–3.73)	0.3249	0.038
	13 to 15	3.23 (1.44–7.25)	0.4108	0.005
	16 or more	2.78 (0.94–8.24)	0.5534	0.066
Bicarbonate		1.00 (0.99–1.02)	0.0082	0.638

**Models with each Level 2 variable**		OR (95%CI)	Standard Error	p-value

Age (years)	6 to 9	1.00	0.5610	
	10 to 12	1.95 (1.03–3.70)	0.3253	0.040
	13 to 15	3.16 (1.42–7.02)	0.4058	0.005
	16 or more	2.74 (0.93–8.14)	0.5530	0.069
Sodium		1.00 (0.98–1.02)	0.0079	0.812

Age (years)	6 to 9	1.00	0.5637	
	10 to 12	1.96 (1.03–3.72)	0.3255	0.039
	13 to 15	3.14 (1.42–6.98)	0.4057	0.006
	16 or more	2.75 (0.93–8.15)	0.5534	0.069
Chlorine		1.00 (0.99–1.01)	0.0044	0.882

Age (years)	6 to 9	1.00	0.5191	
	10 to 12	1.97 (1.04–3.74)	0.3263	0.039
	13 to 15	3.05 (1.38–6.75)	0.4031	0.006
	16 or more	2.73 (0.92–8.10)	0.5532	0.070
Magnesium	0.97 (0.90–1.05)	0.0348	0.416	

Age (years)	6 to 9	1.00	0.5527	
	10 to 12	1.96 (1.03–3.71)	0.3250	0.004
	13 to 15	3.19 (1.43–7.09)	0.4067	0.005
	16 or more	2.74 (0.93–8.13)	0.5525	0.068
pH	0.41 (0.01–16.63)	1.6816	0.614	

Age (years)	6 to 9	1.00	0.5584	
	10 to 12	1.96 (1.03–3.71)	0.3251	0.039
	13 to 15	3.17 (1.42–7.07)	0.4082	0.006
	16 or more	2.75 (0.93–8.17)	0.5532	0.068
Conductivity	1.00 (1.00–1.01)	0.0018	0.885	
